# Effects of neuromuscular training on stability in volleyball athletes: a systematic review and meta-analysis

**DOI:** 10.3389/fspor.2025.1724934

**Published:** 2026-01-12

**Authors:** Fei Yang, Changda Lu, Xin Yun, Chenxiang Qian

**Affiliations:** 1 Department of Physical Education (Baoding), North China Electric Power University, Baoding, Hebei, China; 2 School of Sport Science, Beijing Sport University, Beijing, China

**Keywords:** neuromuscular adaptation, proprioception training, sports injury prevention, stability, volleyball

## Abstract

**Introduction:**

Neuromuscular training (NMT) has been recognized as an effective strategy for improving sensorimotor function and mitigating injury risk in athletic populations. This systematic review and meta-analysis evaluated the effects of NMT on dynamic and static stability in volleyball players. This review assessed potential moderators, including sex and body region.

**Methods:**

Relevant randomized controlled trials published between 1 January 2015, and 30 June 2025, were identified through systematic searches of major electronic databases. The search was limited to English-language publications and was conducted across Web of Science, PubMed, the Cochrane Library, Scopus, ScienceDirect, and PEDro. Following the screening and eligibility assessment, seven studies met the inclusion criteria and were included in the final analysis.

**Results:**

Random-effects meta-analysis indicated that NMT was associated with moderate improvements in overall stability [SMD = 0.63, 95% CI (0.35, 0.9), I2 = 67.5%]. Moderator analyses suggested that benefits were more apparent in the upper limb compared to the lower limb and showed sex specific adaptations: males exhibited greater improvements in upper limb stability, whereas females showed more pronounced effects in the lower limb.

**Discussion:**

Potential optimization mechanisms include enhanced lower-limb biomechanical responses through plyometric training, improved upper-limb kinetic chain synergy, and increased proprioception. NMT induced gender differentiated patterns of stability improvement in volleyball athletes, with males benefiting more from upper-body training while female athletes demonstrated greater adaptability in enhancing lower-body stability.

**Systematic Review Registration:**

https://www.crd.york.ac.uk/prospero/display_record.php?ID=CRD420251146182, PROSPERO CRD420251146182.

## 1 Introduction

The evolution of power- and height-oriented playing styles in modern competitive volleyball has intensified match demands, placing higher demands on athletes' explosive power, dynamic stability, and repetitive jumping ability. Issues such as lower limb asymmetry (1) and ankle instability (2) in professional volleyball players have led to a surge in risks such as anterior cruciate ligament (3) and ankle injuries (4), threatening the healthy development of athletes' careers. As a net-based team sport, competitive volleyball combines high-density competition with a long season of training. This compact schedule necessitates careful management of the training load and recovery periods. Effective training strategies are therefore critical to both individual performance and team success, with neuromuscular control serving as the foundation for skill execution and posture regulation (5).

Neuromuscular Training (NMT) is broadly recognized as an integrative training modality that targets the sensorimotor and neuromuscular systems to optimize motor control, enhance dynamic joint stability, and improve functional movement efficiency (6). By simultaneously stimulating proprioceptive pathways, reinforcing feedforward and feedback neuromuscular mechanisms, and strengthening key musculotendinous structures, NMT contributes to substantial gains in muscular strength, explosive power, postural control, and movement coordination (7). These adaptations improve biomechanical alignment during high-demand tasks such as landing, cutting, and pivoting, thereby reducing modifiable intrinsic risk factors for lower-limb injuries (8). Beyond injury mitigation, NMT has been shown to promote more economical movement patterns and facilitate (9).

The importance of systematically developing and implementing NMT across various sports is underscored by evidence from football, basketball, and other high-intensity team sports (10, 11), where athletes are regularly exposed to complex, multidirectional demands. In football, NMT-based programs have significantly reduced the incidence of anterior cruciate ligament injuries and ankle sprains by improving trunk control (12–14), lower-limb alignment, and reactive stability during unanticipated movements. In basketball, NMT has enhanced jump-landing mechanics, reduced valgus loading, and supported improvements in agility and change-of-direction performance, thereby contributing to both performance enhancement and injury prevention (15–17). Similarly, in adapted sports, NMT serves as a foundational component for improving functional stability, compensatory motor strategies, and overall movement safety in athletes with physical impairments (18). Collectively, findings across these varied athletic contexts highlight NMT as a versatile, evidence-based approach that addresses the neuromuscular and biomechanical demands fundamental to high-level sport.

NMT has become a primary means of preventing injuries and enhancing athletic performance in volleyball players (19). Although NMT has been introduced in volleyball, its use has largely remained within rehabilitation settings. Its proactive application in healthy athletes to enhance neuromuscular control, strengthen dynamic and static balance, improve performance, and reduce injury risk has not been systematically implemented or rigorously evaluated. Existing studies further suffer from heterogeneous samples, single-intervention designs, and limited integration, which constrain the synthesis of clear conclusions (20–23). In addition, current research remains inconclusive regarding the impact of gender on NMT effectiveness. One study (24) emphasizes that a deeper understanding of the role of immutable factors, such as gender, can help refine training programs to optimize muscle mass maintenance and health outcomes. Meanwhile, another study notes that there is ongoing debate over the most effective approach, as factors such as gender, level of play, and program duration significantly influence injury-prevention outcomes (25).

To address the current lack of systematic evidence on NMT in volleyball, this study draws extensively on existing research by reviewing NMT studies on volleyball players published domestically and internationally over the past decade. Experimental studies evaluating intervention effects were excluded, whereas observational studies were screened for the meta-analysis. The results of this study will help clarify the overall effects of NMT on volleyball players' stability, to investigate the underlying mechanisms responsible for the differential effects of NMT across different body parts and between genders, identify the efficacy differences of various training modes, and provide a scientific basis for effectively enhancing volleyball players' training outcomes and preventing injuries.

## 2 Methods

This systematic review was conducted in accordance with the Preferred Reporting Items for Systematic Reviews and Meta-Analyses (PRISMA) 2020 guidelines (26). The completed PRISMA 2020 checklist is available as Supplementary Table S2.

### 2.1 Protocol and registration

The review protocol was prospectively registered on 17 September 2025 in the International Prospective Registry of Systematic Reviews (PROSPERO; ID: CRD420251146182).

### 2.2 Eligibility criteria

Literature selection was conducted in strict accordance with the PICOST framework, considering population, intervention, comparison, outcome, study design, and timeframe, as shown in Table 1. A dual independent review system was implemented for all studies to ensure objectivity and accuracy of the screening process. The final decision for inclusion or exclusion was made by two independent authors based on the inclusion criteria. Disagreements between the reviewers were resolved through discussion with a third-party expert to reach a consensus.

**Table 1:** PICOST framework.

PICOST element	Description
Population (P)	Healthy volleyball players without current musculoskeletal injuries
Intervention (I)	Neuromuscular training programs, including plyometric and sensorimotor-focused exercises
Comparator (C)	Control conditions involving no neuromuscular training or usual volleyball training alone
Outcomes (O)	Dynamic and static stability outcomes assessed using validated clinical or functional measures of upper- and lower-limb stability
Study Design (S)	Randomized controlled trials (RCTs)
Timeframe (T)	Outcomes assessed pre- and post-intervention; eligible studies published between January 1, 2015, and June 30, 2025

Inclusion Criteria: (I) intervention was NMT, (II) the subjects were healthy volleyball players without injuries, (III) outcome measures were dynamic and/or static stability, (IV) the language of publication was English, (V) the study was a randomized controlled trial (RCT). Exclusion Criteria: (I) the intervention in the experimental group was not NMT, (II) subjects were not healthy volleyball players, (III) outcome measures were not related to dynamic or static stability, (IV) non-English literature, (V) the control group participated in the NMT intervention, (VI) prospective or retrospective studies, (VII) books, conference proceedings, and literature reviews, (VIII) duplicate articles, (IX) single-arm studies.

### 2.3 Information sources and search strategy

Two independent reviewers performed double-masked literature searches and screenings. The search encompassed studies published between 1 January 2015, and 30 June 2025, across major databases, was restricted to articles published in English, and was conducted in Web of Science, PubMed, the Cochrane Library, Scopus, ScienceDirect, and PEDro, using keywords such as Neuromuscular training, neuromuscular, Plyometric, proprioceptive, NMT, Volleyball players, Volleyball athletes, team sport, Stability and Balance. The full search strategy for each database is detailed in Supplementary Table S1. In addition to EndNote for reference management, this research used Excel to facilitate the blind screening process and data extraction, ensuring the reproducibility of the study. GraphPad Prism 10 (GraphPad Software, San Diego, California, USA) was used to create a literature screening flowchart.

The search strategy was developed based on the PICOST framework. The complete search string used was: (“Neuromuscular training” OR “Plyometric” OR “NMT”) AND (“Volleyball” OR “Volleyball Players” OR “Volleyball Athletes”) AND (“Stability” OR “Balance”). Additionally, the reference lists of all included articles were manually screened to identify any relevant studies not captured in the electronic database search.

**Figure 1:**
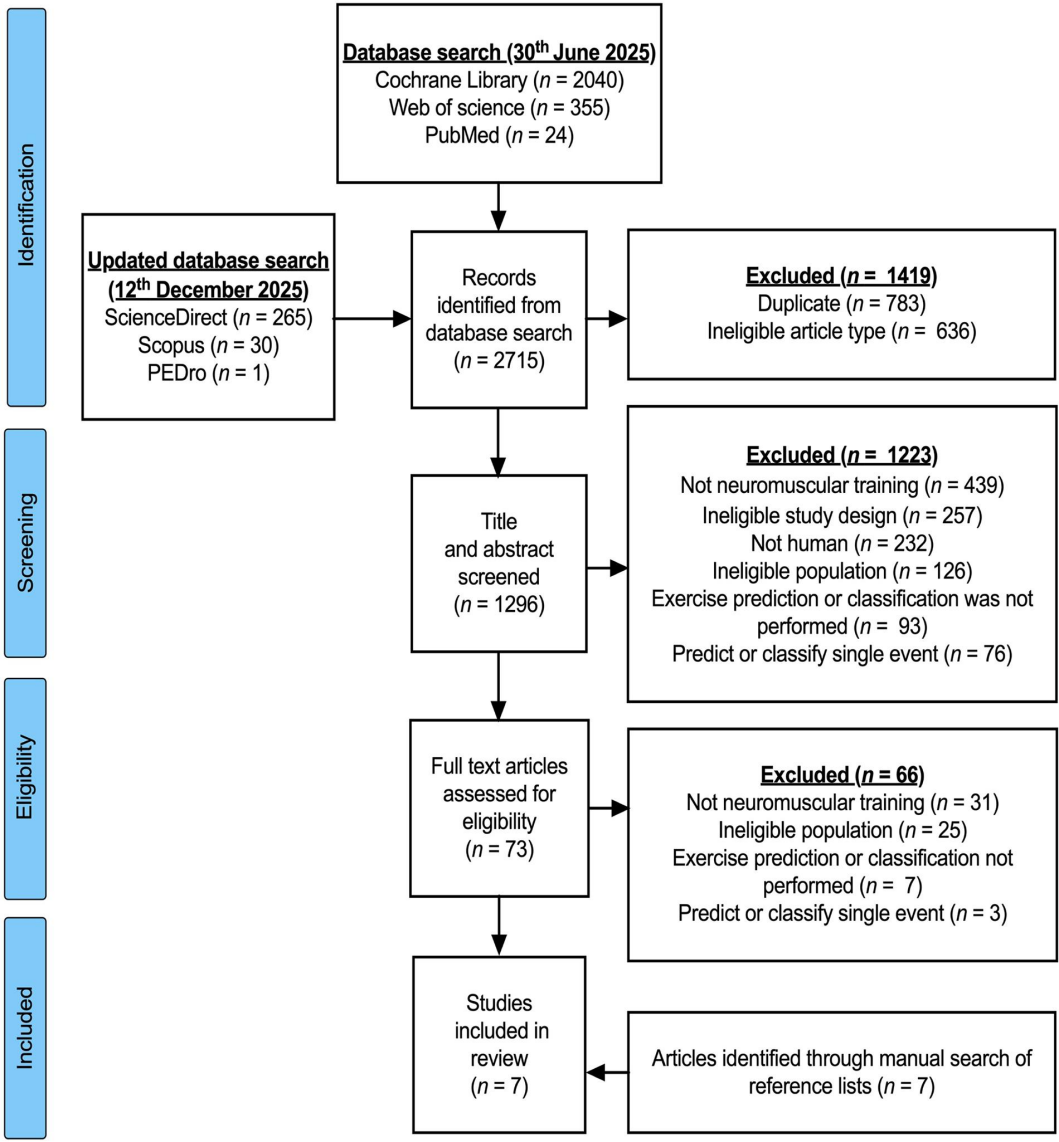
Literature screening and inclusion.

Through the systematic search, a total of 2,715 potentially relevant articles were identified. After removing duplicate records and studies that did not meet the eligibility requirements, 1,296 articles proceeded to the title and abstract screening stage, of which 1,223 were excluded for not aligning with the research topic. Full-text assessment was then conducted for the remaining 73 articles. Of these, 66 were excluded for failing to meet the inclusion criteria, including inappropriate exercise intervention protocols, the absence of a control group, and study populations that were not volleyball athletes or outcome measures that were not relevant or valid. Ultimately, seven intervention studies were included in the meta-analysis, as shown in Figure 1.

### 2.4 Data extraction

Two authors (FY and CQ) independently screened and selected eligible studies, resolving discrepancies through discussion and consensus. For all studies meeting the inclusion criteria, the primary outcome was balance ability. Secondary outcomes, including postural sway, center of pressure displacement, stability index, and reaction time during balance tasks.

### 2.5 Data items

Covariates included the first author, publication year, sample size, implemented interventions, and relevant outcome measures, which were systematically extracted from each study. For studies with incomplete data, the corresponding authors were contacted via email for Supplementary Information, or data were deeply analyzed and extracted from the existing literature.

### 2.6 Risk of bias of individual studies

The methodological quality of the included studies was comprehensively evaluated according to the Cochrane Handbook for Systematic Reviews of Interventions (version 5.1.0) for RCTs. The evaluation items included the method of random sequence generation, implementation of allocation concealment, blinding of participants and personnel, blinding of outcome assessment, completeness of outcome data, selective reporting, and other potential sources of bias. The Cochrane Risk of Bias tool (Review Manager 5.4, Cochrane, London, U.K.) was used for quality assessment.

### 2.7 Summary measures

As the included studies used continuous outcome measures and differed in testers, equipment, measurement methods, and units, the standardized mean difference was chosen as the effect size to reduce the impact of between-study differences. Point estimates and 95% confidence intervals (95% CI) are reported. Effect sizes were categorized according to Cohen's standards (27): ≤0.2 (trivial), 0.2–0.5 (small), 0.5–0.8 (medium), and ≥0.8 (large). The statistical significance level for the meta-analysis was set at *p* < 0.050. The stability of the results was assessed by comparing the differences between the random- and fixed-effects models.

### 2.8 Assessment of heterogeneity

Statistical analyses were performed using Stata 15.0 and Stata 18.0 (StataCorp LLC, Lakeway Drive, College Station, USA). The statistical model (fixed or random effects) was chosen based on the heterogeneity analysis. Heterogeneity was tested using the Q test (*χ*^2^ test) with *α* *=* 0.100. If *p* < *α*, heterogeneity was considered to be present; if *p* ≥ *α*, homogeneity was assumed. The degree of heterogeneity was quantified using the *p*-value and the *I*^2^ statistic (ranging from 0% to 100%). If *I*^2^ ≤ 50% and *p* > 0.100, a fixed-effects model was used for the Meta-analysis; if *I*^2^ > 50% or *p* < 0.100, a random-effects model was used. To explore potential sources of heterogeneity in the study results, subgroup analyses were conducted by stratifying outcomes by different study characteristics or intervention conditions to assess the influence of relevant factors on overall effect estimates.

### 2.9 Risk of bias across studies and sensitivity analyses

Funnel plot analysis was used to detect and assess potential publication bias. Sensitivity analyses were conducted to evaluate the robustness of the pooled effect estimates by sequentially removing individual studies (leave-one-out analysis) and re-estimating the overall effect. All analyses were performed using Stata 15.0 (StataCorp LLC, Lakeway Drive, College Station, USA). The certainty of evidence for each outcome was assessed qualitatively by considering risk of bias, consistency of results, and robustness of the pooled estimates.

## 3 Results

### 3.1 Study characteristics

A total of 7 randomized controlled trials were included in this systematic review, all of which investigated the effects of NMT- based interventions on stability-related outcomes in volleyball players. The studies were published between 2016 and 2024 and were conducted in Turkey, Iran, Serbia, and Tunisia. Overall, the included studies ranged in sample size from 24 to 66 participants, comprising both male and female volleyball players across adolescent and young adult age groups.

All experimental interventions were grounded in NMT principles. The training content included dynamic and static stability exercises for the upper and lower limbs, respectively, with training protocols incorporating various combinations of balance, proprioceptive, plyometric, perturbation, elastic-band, and neuromotor exercises. Training programs primarily focus on improving neuromuscular control, including balance, proprioception, and plyometric training. Intervention durations ranged from 6 to 8 weeks, with training frequencies of 2 to 3 sessions per week. In several studies, NMT was implemented alongside regular volleyball training, whereas others integrated NMT elements into warm-up routines. The control group received conventional volleyball training, traditional strength training, or no intervention. Outcome measures assessed the dynamic and static stability of the upper and lower limbs using tests such as the Y Balance Test and Dynamic Postural Stability Index. The basic characteristics of the included studies are presented in Table 2.

**Table 2:** Basic characteristics of included studies.

Study reference (Year of study publication)	Country	Study type	Sample size	Age (yrs)	Time of training program	Outcome measures	Final conclusion
(49)	Turkey	RCT	EG = F17 CG = F17	15.5 ± 0.9	EG: 3 sessions/wk * 6 wks Plyometric + regular volleyball training. CG: Regular volleyball training.	SEBT	Six-week plyometric training enhanced non-dominant limb balance and single-leg hop performance in female volleyball players, with no additional improvement in the hamstring–quadriceps ratio compared with standard training.
(28)	Iran	RCT	EG = F16 CG = F16	18–24	EG: 3 sessions/wk * 6 wks EFIE. CG: Regular volleyball training.	DPSI	External focus instruction exercise can significantly improve landing kinetics and functional performance, and reduce the risk of ACL injury in female volleyball athletes.
(19)	Serbia	RCT	EG = F32 CG = F34	11.1 ± 0.7	EG: 2 sessions/wk * 8 wks NMT and volleyball training. CG: Regular volleyball training	KTK	An 8-week neuromuscular training program significantly enhanced motor competence and improved agility and vertical jump performance in young female volleyball players compared with regular volleyball training alone.
(29)	Tunisia	RCT	EG = M14 CG = M13	14.3–15.4	8 wks; 2×/week; 45 min/session (15 min warm-up + 30 min EBT)	CoP	Eight weeks of elastic-band training significantly improved 1RM, CMJ and SLJ and anteroposterior balance in pubertal male volleyball players; RSI and other balance measures showed no between-group differences.
(30)	Iran	RCT	EG = M15 CG = M15	16.6 ± 2.2	EG: 3 sessions/wk * 8 wks. CG: Usual warm-up.	YBT-LQ, YBT-UQ	Verhagen NEMEX training improved hopping, lower-quarter Y-balance, and Davies tests in high-risk volleyball players; no significant effect on FMS or upper-quarter Y-balance. Recommended as a warm-up to reduce injury risk.
(31)	Iran	RCT	EG = M12 CG = M12	24.4 ± 2.2	EG: 3 sessions/wk * 6 wks Perturbation training. CG: Regular training.	YBT-UQ	Perturbation training improved rotator cuff strength, shoulder proprioception, and upper extremity performance in male volleyball players.
(56)	Turkey	RCT	EG = M13 CG = M13	18–23	EG: 3 sessions/wk * 8 wks Neuromotor Training. CG: Regular training.	CKCUEST	Neuro-athletic training significantly improves flexibility, serve speed, and upper limb stability in elite volleyball players, outperforming traditional training.

EG, Experimental Group; CG, Control Group; M, Male; F, Female; wks, weeks; Cop, Center of Pressure; YBT-LQ, Y Balance Test Lower Quarter; YBT-UQ, Y Balance Test Upper Quarter; KTK, Körperkoordinationstest für Kinder (Body Coordination Test for Children); SEBT, Star Excursion Balance Test; CKCUEST, Closed Kinetic Chain Upper Extremity Stability Test; DPSI, Dynamic Postural Stability Index; EFIE, External Focus Instruction Training; EBT, Elastic Band Training.

### 3.2 Risk of bias assessment

**Figure 2:**
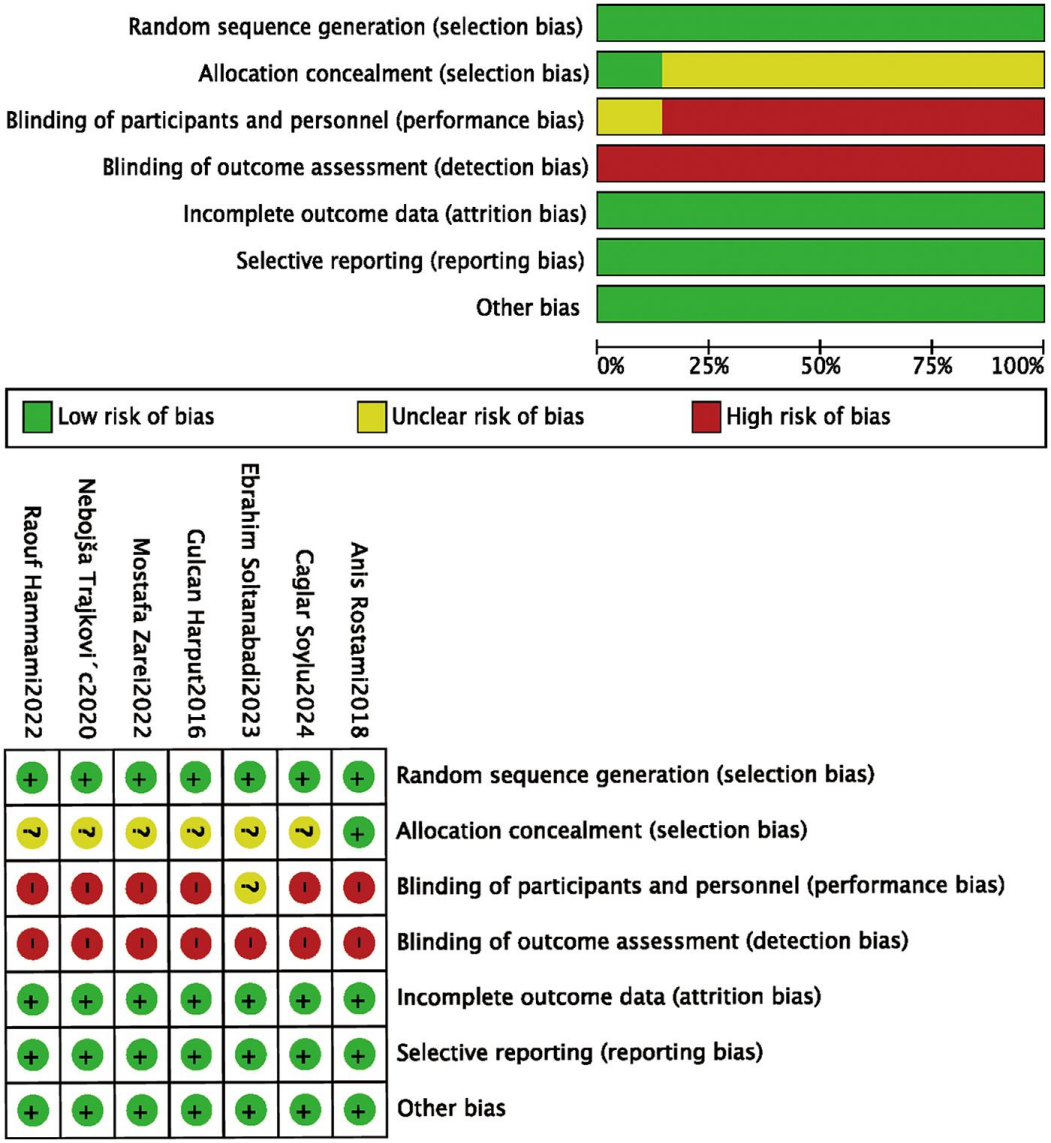
Risk of bias graph and risk of bias summary.

The methodological quality of the included studies was assessed using the Cochrane Risk of Bias tool, indicating an overall good quality, as shown in Figure 2. All 7 included studies were RCTs. Regarding allocation concealment, only one study clearly described the specific method used, whereas others provided no relevant information. Owing to ethical requirements for human studies, all studies obtained informed consent before the experiment, limiting the implementation of double-blinding. All studies reported complete outcome data, with no missing data or attrition bias. All studies reported complete outcome data, with no evidence of missing data, participant attrition, or differential dropout between groups, indicating a low risk of attrition bias. In addition, the reported outcomes were consistent with the study objectives, and no selective reporting was identified. Overall, the methodological quality of the included studies was good, but the reporting transparency concerning allocation concealment and blinding implementation requires further improvement.

### 3.3 Sensitivity analysis

**Figure 3:**
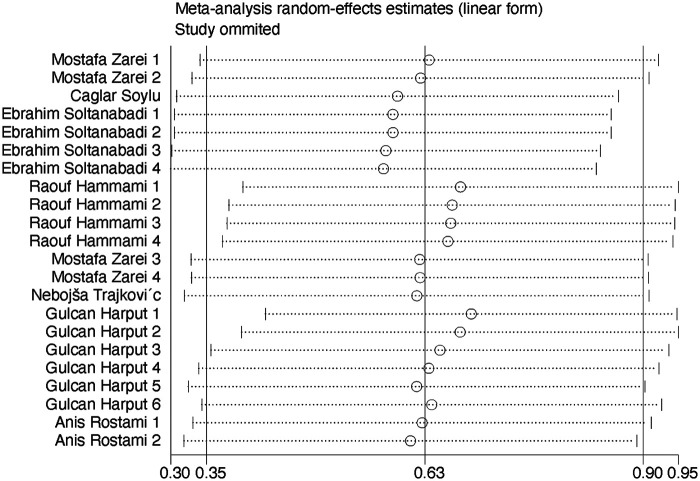
Sensitivity analysis of NMT's effect on dynamic and static stability in volleyball players.

Sensitivity analysis was performed on the 22 included studies. Results are as follows. As shown in Figure 3, removing any single study did not significantly alter the overall effect size (no extreme outliers observed), indicating relatively robust results. However, some studies may have affected the precision of the overall result. Further analysis of methodological differences is warranted.

### 3.4 Overall effect of NMT on stability

**Figure 4:**
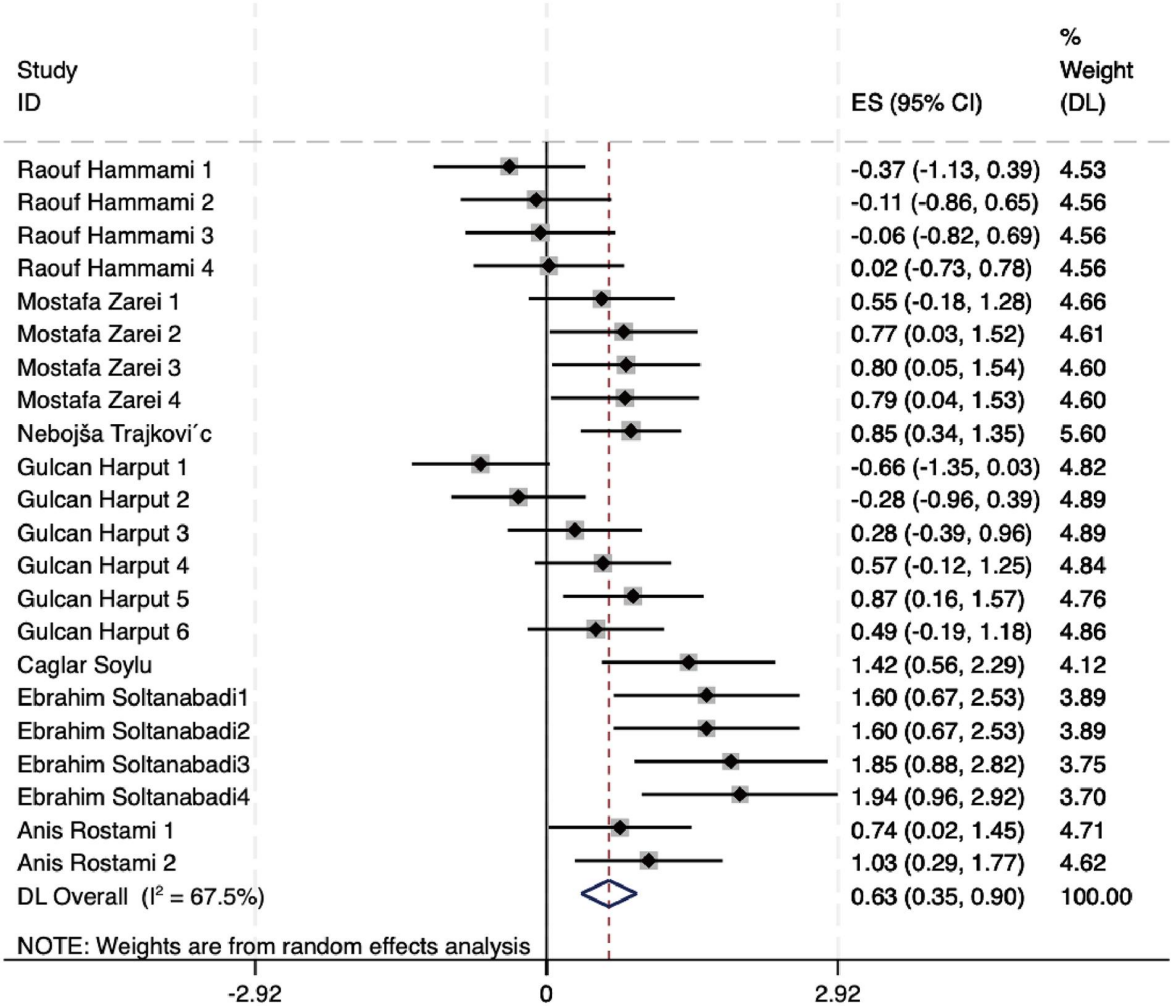
Forest plot of meta-analysis on NMT's effect on dynamic and static stability in volleyball players.

The 7 included articles (22 studies) showed heterogeneity (*I*^2^ = 67.5%; *Q*-test, *p* < 0.100), indicating moderate to high between-study variability. Consequently, a random-effects model was deemed appropriate and applied for the pooled analysis. The observed heterogeneity was most likely attributable to methodological and clinical differences across studies, particularly variations in limb measurement sites (upper limb vs. lower limb), outcome assessment tools, and intervention components. Given this anticipated variability, subgroup analyses based on upper- and lower-limb stability assessments were planned and conducted further to explore potential sources of heterogeneity, as shown in Figure 4. When all 22 effect sizes were synthesized using a random-effects model, NMT interventions demonstrated a statistically significant overall beneficial effect on stability outcomes compared with control conditions. The pooled standardized effect size was 0.63 (95% CI: 0.35 to 0.90) and was statistically significant (*p* < 0.001), indicating a moderate positive effect of NMT on dynamic and static stability in volleyball players. The overall effect estimate favored the NMT groups, with the confidence interval not crossing the line of no effect, supporting the robustness of the pooled result.

### 3.5 Subgroup analysis

**Figure 5:**
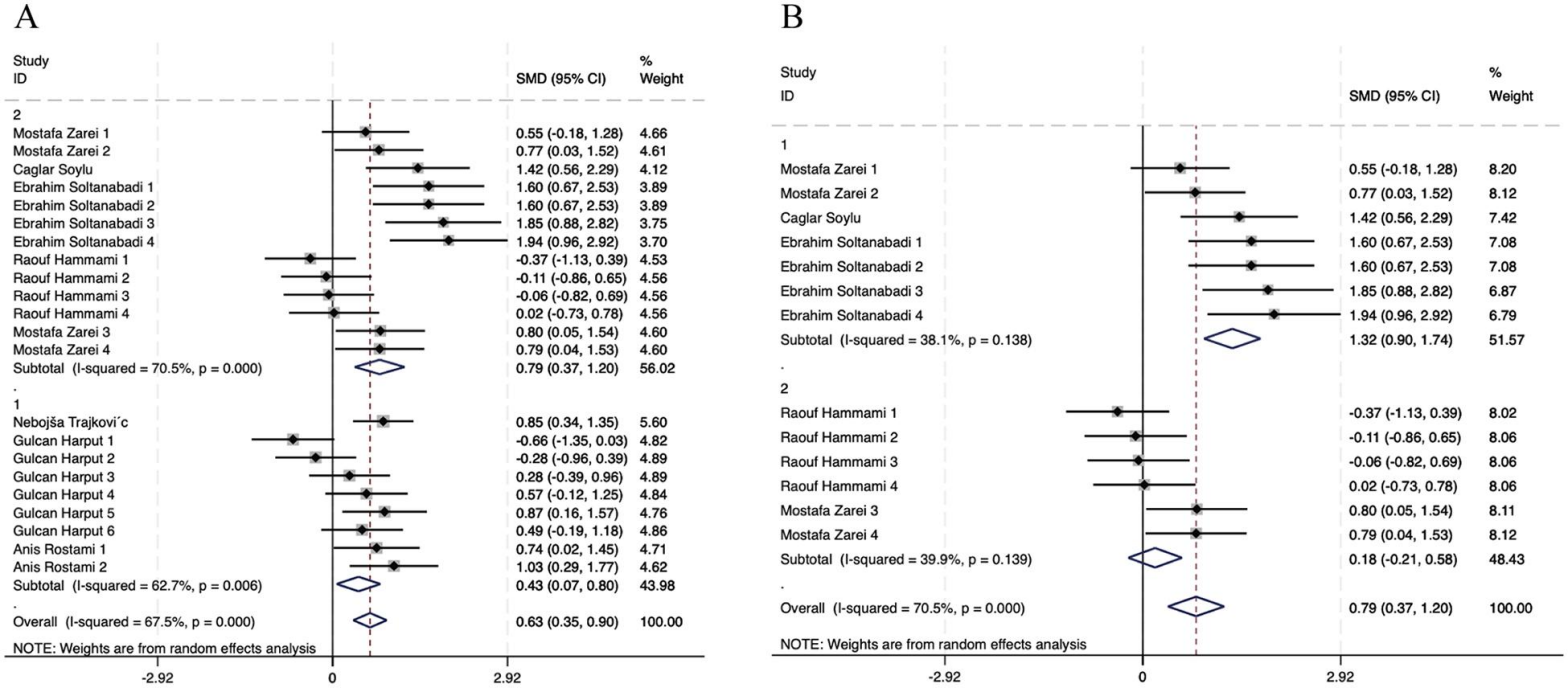
Subgroup analyses for stability in volleyball players. **(A)** Comparison by Gender, **(B)** Comparison by Limb for males.

Subgroup analyses were conducted to further explore potential sources of heterogeneity and to examine whether the effects of neuromuscular training on stability differed by participant sex and the anatomical site of outcome assessment. A total of 22 experimental comparisons were included in these analyses. As illustrated in Figures 5A,B, studies were first stratified by sex, and a further subgroup analysis was performed among male athletes based on the body region assessed.

Based on the subgroup analysis, moderate between-group heterogeneity was observed (67.5%). Within-group heterogeneity was also moderate overall (*I*^2^ = 62.7%). These findings suggested that variability in study characteristics contributed meaningfully to the observed heterogeneity. Subsequent analyses using a random-effects model indicated that differences in the anatomical site of stability assessment were a likely source of this variability.

Among male volleyball players, subgrouping by limb site yielded clearly differentiated results. For upper limb stability, the analysis of 7 studies demonstrated a large, statistically significant effect size of 1.32 (95% CI: 0.90, 1.74; *p* < 0.001), with low within-group heterogeneity (*I*^2^ = 38.1%). In contrast, for lower limb stability, pooling six studies yielded a small, non-significant effect size of 0.18 (95% CI: −0.21, 0.58; *p* = 0.369), with low heterogeneity (*I*^2^ = 39.9%). This confirms that NMT significantly improves upper-limb stability in males, and the variation in limb measurement sites is a primary source of heterogeneity.

Sex based subgroup analyses further confirmed the overall effectiveness of NMT. The synthesis of nine studies on female athletes showed a medium, statistically significant effect (ES = 0.43, 95% CI: 0.07, 0.80; *p* = 0.020), despite moderate heterogeneity (*I*^2^ = 70.5%). Similarly, pooling 13 studies on male athletes yielded a medium-to-large, highly significant effect (ES = 0.79, 95% CI: 0.37, 1.20; *p* < 0.001). In summary, NMT is an effective intervention for enhancing stability in both male and female volleyball players. Interventions targeting the upper limbs of male athletes yield greater effects than those targeting the lower limbs. Sex based subgroup analyses further confirmed the overall effectiveness of NMT.

### 3.6 Effect of NMT on stability by limb part

**Figure 6:**
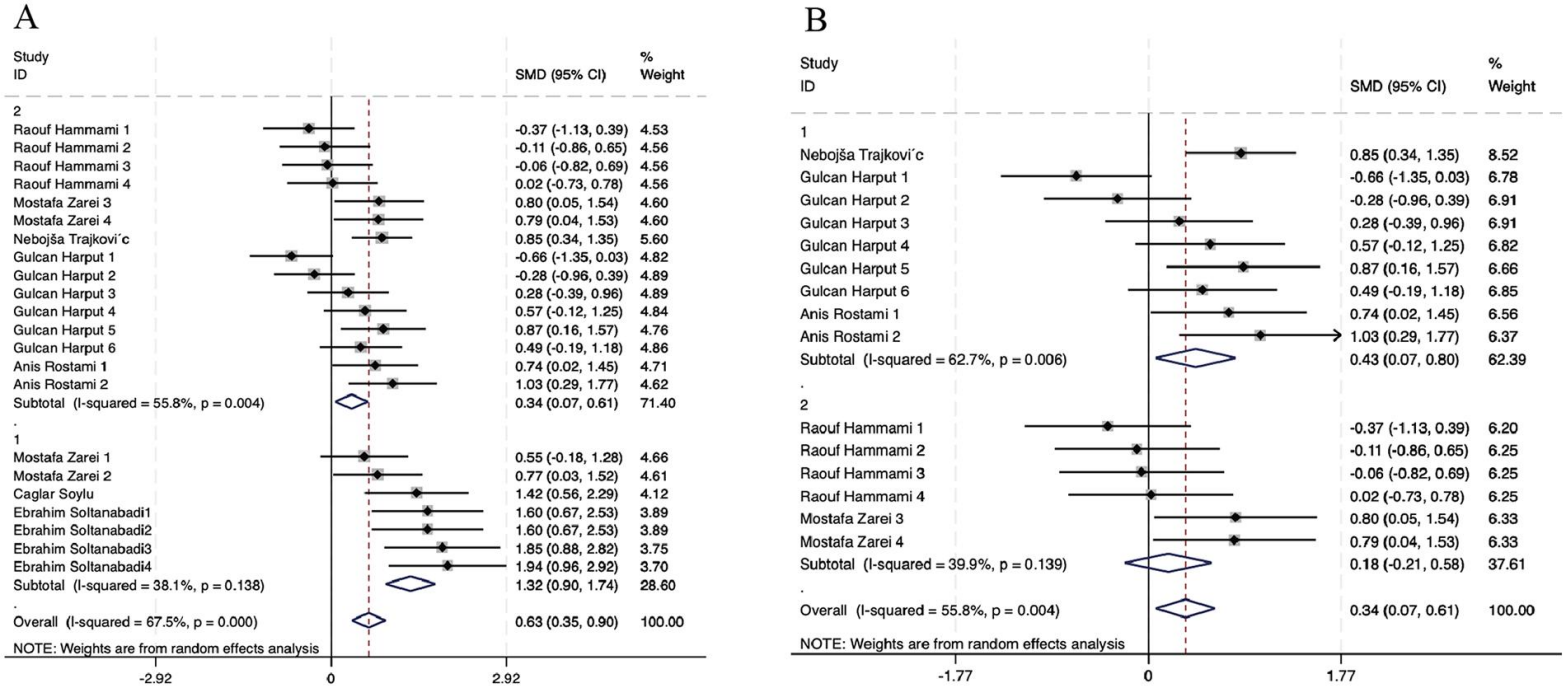
Subgroup analyses for stability in volleyball players. **(A)** Comparison by Limb, **(B)** Comparison by lower limb for Gender.

To further clarify the differential effects of NMT on stability, the 22 experimental comparisons were stratified by anatomical region and analyzed separately. The corresponding forest plots are presented in Figures 6A,B.

Within-group heterogeneity was low for upper-limb stability (*I*^2^ = 38.1%, *p* = 0.138). Pooling seven studies showed a large effect size of 1.32 (95% CI: 0.90, 1.74) and significant (*p* < 0.001), confirming that NMT significantly enhances upper limb stability in male and female volleyball players. within-group heterogeneity was slight for lower-limb stability (*I*^2^ = 55.8%, *p* = 0.004). Pooling 15 studies yielded a medium effect size of 0.34 (95% CI: 0.07, 0.61), significant (*p* = 0.015 < 0.05), suggesting that NMT is also effective in enhancing lower-limb stability across male and female volleyball players, although the magnitude of the effect was smaller than that observed for upper-limb outcomes.

To further explore potential sex-related differences in lower-limb stability outcomes, an additional subgroup analysis by gender was conducted using a random-effects model, as shown in Figure 6. For female lower-limb stability, within-group heterogeneity was moderate (*I*^2^ = 62.7%, *p* = 0.006). Pooling nine studies showed a medium effect size of 0.43 (95% CI: 0.07, 0.80), significant (*p* = 0.020), indicating that NMT significantly enhances lower-limb stability in female volleyball players. For male lower limb stability, within-group heterogeneity was low (*I*^2^ = 39.9%, *p* = 0.139). Still, pooling six studies yielded a small, non-significant effect size of 0.18 (95% CI: −0.21, 0.58; *p* = 0.369), possibly due to limited sample sizes or random error. This confirms that NMT's improvement effect on lower-limb stability is superior in females than in males. Highlighting the importance of considering both limb-specific and sex-specific factors when designing and implementing NMT programs.

### 3.7 Publication bias results

**Figure 7:**
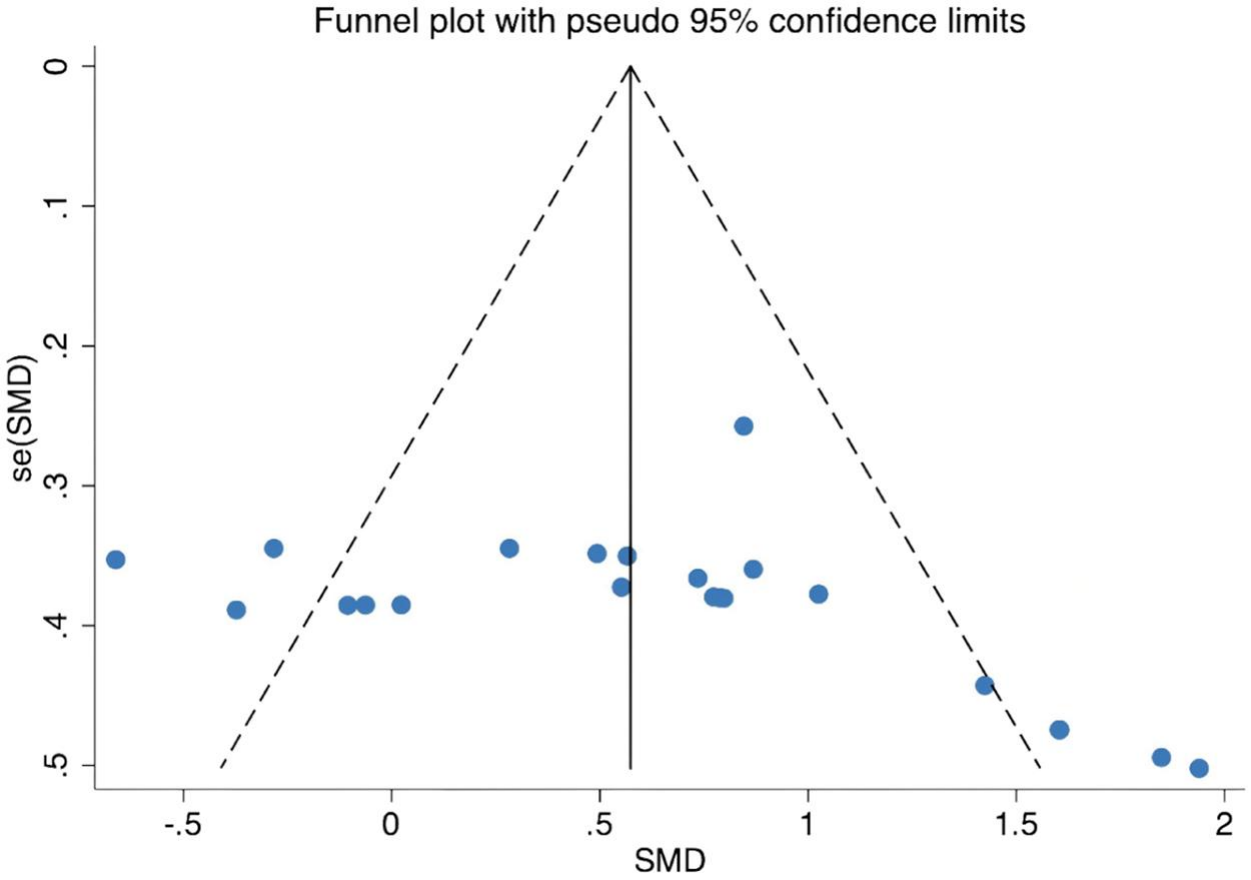
Funnel plot for publication bias.

The funnel plot for the 22 included effect sizes derived from seven RCTs is presented in Figure 7. Visual inspection revealed an approximately symmetrical distribution of studies around the overall pooled effect, and *p* = 0.176 suggested no significant publication bias, suggesting a low likelihood that the observed results were influenced by selective publication. Taken together, the symmetrical funnel plot and non-significant test for asymmetry support the robustness of the present findings and indicate that the overall conclusions regarding the beneficial effects of NMT on stability in volleyball players are unlikely to be substantially affected by publication bias.

## 4 Discussion

### 4.1 Effects and mechanisms of NMT on stability

The results of the meta-analysis indicate that NMT significantly enhances dynamic and static stability in volleyball players. This aligns with existing research suggesting that NMT, by integrating balance, proprioception, and plyometric training, effectively enhances neuromuscular control, thereby improving postural stability and dynamic joint control during movement (32). The specific mechanisms include three main aspects. NMT promotes lower limb biomechanical optimization. Plyometric training can improve knee valgus moments during landing and reduce the risk of ACL injury (33). NMT enhances upper-limb kinetic chain synergy (34). Elastic band resistance training strengthens the rotator cuff and core stability and improves spiking action control (28). The NMT reinforces proprioception (35). Balance training stimulates plantar pressure receptors and enhances dynamic postural stability (30).

Different NMT methods were applied to other body parts. Comprehensive training yields optimal results by simultaneously activating multi-joint synergy and control (36). Pure plyometric training has limited improvement in lower limb stability, possibly because traditional volleyball training already partially addresses lower limb strength foundations (37).

Understanding the differences between various NMT methods is essential for effectively improving training outcomes. Some research suggests that an 8-week elastic band resistance training program improves jump performance and other volleyball-related parameters in young male athletes and should be considered as a supplement to regular volleyball training (29, 38–40). Volleyball players at high risk of continuous injuries can prevent injuries by adding NEMEX training to their regular training (30). Specific NMT requires rigorous standards; otherwise, ideal training effects cannot be achieved. Eccentric overload flywheel resistance training is superior to traditional strength training in developing maximal strength, increasing muscle hypertrophy, and enhancing neural adaptation (41). Arranging PNF stretching before core stability training leads to better neuromuscular adaptation training effects (42). Avoid performing other types of landing exercises during plyometric training sessions, as impaired postural control increases the probability of lower-limb injury (43). When using the Star Excursion Balance Test, directions with relatively high sensitivity are chosen to save time and improve statistical accuracy (44). An integrative NMT program lasting 16–20 min, 2–3 times per week, for 4–6 months can achieve optimal sports injury-prevention effects (45).

### 4.2 Effects of NMT on different limbs

Meta-regression and subgroup analyses confirmed that the limb part was a major source of heterogeneity. Subgroup analysis by limb showed that NMT significantly enhanced upper-limb stability and effectively enhanced lower-limb stability. The significant improvement in upper limb stability may be attributed to specialized training targeting the shoulder joint and core muscles within the NMT, which directly strengthens the upper limb kinetic chain synergy (28). The smaller effect size for lower limb stability may reflect the fact that traditional volleyball training already partially covers lower limb strength and balance, leaving less marginal gain for NMT (19). Additionally, differences in the sensitivity of lower-limb test measures across studies could contribute to the variation in results (46). NMT plyometric methods improved balance in the nondominant leg (36, 47–49). The reason might be that the dominant leg is exercised more frequently, develops better, has better specific performance, and, consequently, better balance (50). The non-dominant leg is less exercised and has poorer balance. Plyometric training promotes the development of the nondominant leg, thereby improving balance (51). The expected effect is an improvement in bilateral balance ability, enhanced balance control during movement, and reduced imbalance during technical actions, thereby lowering injury risk, helping athletes maintain good competitive conditions, and prolonging their careers (51, 52).

### 4.3 Effects of NMT by gender

NMT is an effective means of overcoming lower-limb functional asymmetry and improving functional stability and skill in athletes (53–55). Subgroup analysis showed that NMT's effect on upper limb stability was greater in males than in females. In contrast, its impact on lower limb stability was greater in females than in males. This research adopted different NMT approaches for athletes of different genders. Male Athletes: Implement upper limb perturbation training 3 times each week, combined with closed-chain exercises to strengthen scapular stability (56). Female Athletes: Increase single-leg balance training and neuromotor training to optimize lower-limb force alignment (19). A training duration of at least eight weeks is recommended to consolidate neural adaptations, as supported by evidence from long-term integrative NMT interventions (57).

The significant upper-limb effect in males may be related to the high demand for shoulder stability during actions such as spiking and blocking (56). Studies on isokinetic eccentric training (IET) in males show that IET can improve the strength balance of shoulder internal/external rotators, maintain shoulder dynamic stability, and improve neuromuscular control, potentially reducing injury risk (58). NMT improves flexibility, serve speed, and upper limb performance in elite male volleyball players, demonstrating its effectiveness (56). Perturbation training benefits volleyball players by improving rotator cuff strength, modulating shoulder proprioception, and enhancing upper-limb performance (31). The prominent lower limb effect in females can improve athletic performance and reduce injury risk in female athletes; similar results have been reported in female soccer players (59). This aligns with the biomechanical characteristics of females who have a higher ACL injury risk (60, 61), as NMT improves quadriceps-hamstring strength balance and reduces knee valgus angles. Individual studies have reported that an eight-week NMT program significantly improved motor abilities and physical fitness in female volleyball players aged 10–12 years (19). Plyometric training can enhance performance in female volleyball players (49).

### 4.4 Practical implications

The study found that NMT aimed at enhancing volleyball players' performance primarily focused on improving dynamic stability, emphasizing the development of balance ability during athletic states, enhancing stability of movement postures, boosting performance in sports contexts, and maximizing injury prevention. Emphasizing dynamic NMT methods aligns with the characteristics of volleyball. Although static stability training within the NMT is less common, it can supplement conventional training methods.

Training prescriptions should be gender specific. For male athletes, the focus is on upper-body stability, incorporating upper-limb perturbation training and closed-chain drills to strengthen the scapula. For female athletes, the emphasis shifts to the lower limbs, utilizing single-leg balance and neuromotor training to improve force alignment. A minimum duration of eight weeks is recommended to solidify neural adaptations.

Method selection is critical. Comprehensive training integrating balance and plyometrics yields superior results by activating multi-joint coordination. In contrast, pure plyometric training offers limited gains for lower-limb stability, which may already be partially addressed by conventional volleyball practice. Specific techniques, such as elastic band resistance training or eccentric overload flywheel training, are effective supplements. However, NMT requires precision; for instance, plyometric sessions should avoid additional landing drills to reduce injury risk, and protocols must be followed rigorously.

NMT has broad prospects for future applications. NMT can not only be used to develop dynamic and static abilities in volleyball players but also to assess, diagnose, and screen healthy athletes' abilities. It has broad prospects for selecting elite athletes, improving selection success rates, diagnosing sports injuries, preventing injuries, and aiding in sports rehabilitation (19). Coaches and researchers can use related tests to evaluate athletes' abilities, monitor their training process, accurately assess their physical state and adaptation to training stimuli, and further optimize training programs to enhance athletic performance.

In summary, this meta-analysis provided evidence-based support for the application of NMT in volleyball. Future high-quality RCTs are needed to explore personalized training programs and biomechanical mechanisms.

### 4.5 Limitations

Although this study followed PRISMA guidelines, several limitations should be noted. The included studies generally maintained high methodological rigor, with clearly defined interventions and control conditions, complete reporting of outcomes, and objective primary outcome measures that mitigated detection bias. Nevertheless, certain limitations remain. Only one study explicitly reported allocation concealment, and double-blinding was not feasible due to ethical considerations, which may introduce potential selection or performance bias. Despite these minor limitations, the consistency in study design, intervention protocols, and outcome measures across trials supports the meta-analysis's internal validity. It indicates that the synthesized results reliably reflect the effects of NMT on stability in volleyball players. However, the narrow age range of participants limits the generalizability of the findings, as training effects in adolescents and young adults who are still undergoing physical and psychological development may differ from those observed in older adults. Further research is therefore needed to validate these findings in a broader age range.

In addition, incomplete reporting of blinding and allocation concealment in some studies raises the risk of performance bias. The short intervention period (6–8 weeks) and reliance on immediate post-intervention testing further limit the ability to conclude, leaving the long-term efficacy of NMT and its influence on subsequent performance unclear. Future research should therefore include broader age groups, ensure rigorous methodological reporting, and directly compare different NMT protocols to better determine their specific effects on lower limb stability.

## 5 Conclusions

This meta-analysis demonstrated that NMT significantly improves dynamic and static stability in volleyball players by enhancing lower-limb biomechanics, upper-limb kinetic chain function, and proprioceptive control. Subgroup analyses showed gender- and limb-specific effects, with males benefiting more from upper-limb stability and females from lower-limb stability. These findings provide empirical evidence for group-specific neuromuscular adaptations and clarify methodological factors, such as limb site and outcome measures, that influence study results. Overall, the study offers a scientific basis for developing targeted NMT programs in volleyball and highlights the need for high-quality, long-term research to optimize training strategies for performance enhancement and injury prevention.

## Data Availability

The original contributions presented in the study are included in the article/Supplementary Material, further inquiries can be directed to the corresponding author.
