# Supplementary material for: Effects of neuromuscular training on stability in volleyball athletes: a systematic review and meta-analysis
Source: Front Sports Act Living. 2026 Jan 12;7:1724934. doi: 10.3389/fspor.2025.1724934 (PMC12832796; doi:10.3389/fspor.2025.1724934)
Supplement: Supplementary file 1 [file Table1.docx]

# S1. Search strategies for all databases

| **Database** | **Search strategy** |
| --- | --- |
| PubMed | ("neuromuscular training" OR "plyometric exercise" OR plyometrics OR NMT) AND ("volleyball" OR "volleyball players" OR "volleyball athletes") AND ("postural Balance" OR balance OR stability) |
| Web of Science | TS = (neuromuscular* OR plyometric* OR "balance training" OR sensorimotor* OR proprioceptive training OR "injury prevention") AND TS=(volleyball*) |
| Cochrane Library | (plyometric* OR neuromuscular OR proprioceptive OR jump*) AND (volleyball OR "team sport*" OR athlete*) |
| PEDro | neuromuscular training volleyball balance; also searched: plyometric volleyball balance; neuromuscular training volleyball stability |
| Scopus | TITLE-ABS-KEY ("neuromuscular training" OR plyometric* OR NMT) AND TITLE-ABS-KEY (volleyball OR "volleyball players" OR "volleyball athletes") AND TITLE-ABS-KEY (balance OR stability) |
| ScienceDirect | ("neuromuscular training" OR plyometric* OR NMT) AND (volleyball OR "volleyball players") AND (balance OR stability) |
